# Genetic Parameters, Linear Associations, and Genome-Wide Association Study for Endotoxin-Induced Cortisol Response in *Holstein heifers*

**DOI:** 10.3390/ani15131890

**Published:** 2025-06-26

**Authors:** Bruno A. Galindo, Umesh K. Shandilya, Ankita Sharma, Flavio S. Schenkel, Angela Canovas, Bonnie A. Mallard, Niel A. Karrow

**Affiliations:** 1 Department of Animal Biosciences, University of Guelph, Guelph, ON N1G 2W1, Canada or bruno@uenp.edu.br (B.A.G.); ankitasharma21@gmail.com (A.S.); acanovas@uoguelph.ca (A.C.); 2 Cornélio Procópio Campus, State University of the Northern Parana, Cornélio Procópio 86300-000, PR, Brazil; 3 Department of Pathobiology, University of Guelph, Guelph, ON N1G 2W1, Canada; bmallard@ovc.uoguelph.ca

**Keywords:** heifers, stress, cortisol, GWAS

## Abstract

Lipopolysaccharide (LPS) endotoxin is a well-characterized microbe-associated molecular pattern (MAMP) that forms the outer membrane of both pathogenic and commensal Gram-negative bacteria. It plays a crucial role in triggering inflammatory disorders such as mastitis, acidosis, and septicemia. In heifers, an LPS challenge induces a dynamic stress response, marked by elevated cortisol levels, increased body temperature, and altered immune function. Research indicates that LPS administration leads to a significant rise in cortisol post-challenge. Building on this understanding, the present study aimed to estimate genetic parameters for serum cortisol response to LPS challenge in *Holstein heifers* and its linear associations with production, health, reproduction, and conformation traits. Additionally, a genome-wide association study (GWAS) was conducted to identify genetic regions associated with cortisol response. A total of 252 animals were evaluated for cortisol response, with correlations estimated between cortisol levels and 55 genomic breeding values for key traits. Genetic parameters and heritability for cortisol response were estimated using Residual Maximum Likelihood (REML) in the Blupf90+ v 2.57 software. Single-Step GWAS (ssGWAS) employing a 10-SNP window approach and 42,123 SNP markers was performed to identify genomic regions that explained at least 0.5% of additive genetic variance. Finally, candidate genes and QTLs located 50 kb up and downstream of those windows were identified. The cortisol response showed significant but weak linear associations with cystic ovaries, body maintenance requirements, lactation persistency, milk yield, and protein yield (*p*-value ≤ 0.05) and showed suggestive weak linear associations with udder texture, clinical ketosis, heel horn erosion, and milking speed (*p*-value ≤ 0.15). Cortisol response showed significant additive genetic variance, along with moderate heritability of 0.26 (±0.19). A total of 34 windows explained at least 0.5% of additive genetic variance, and 75 QTLs and 11 candidate genes, comprising the genes *CCL20*, *DAW1*, *CSMD2*, *HMGB4*, *B3GAT2*, *PARD3*, *bta-mir-2285aw*, *CFH*, *CDH2*, *ENSBTAG00000052242*, and *ENSBTAG00000050498*, were identified. The functional enrichment analysis allowed us to infer two instances where these gene products could interfere with cortisol production: the first instance is related to the complement system, and the second one is related to the EMT (Epithelium–Mesenchymal Transition) and pituitary gland formation. Among the QTLs, 13 were enriched in the dataset, corresponding to traits related to milk (potassium content), the exterior (udder traits, teat placement, foot angle, rear leg placement, and feet and leg conformation), production (length of productive life, net merit, and type), and reproduction (stillbirth and calving ease). In summary, the cortisol response to LPS challenge in *Holstein heifers* seems to be moderately heritable and has weak but significant linear associations with important production and health traits. Several candidate genes identified could perform important roles, in at least two ways, for cortisol production, and QTLs were identified close to regions of the genome that explained a significant amount of additive genetic variance for cortisol response. Therefore, further investigations are warranted to validate these findings with a larger dataset.

## Simple Summary

This study investigated the genetic basis of cortisol response to immune stress induced by bacterial endotoxin in 252 *Holstein heifers*. Cortisol response showed significant additive genetic variance, along with moderate heritability (h^2^ = 0.26) and significant but weak linear associations with key traits, including milk yield, protein yield, and cystic ovaries. A genome-wide association study revealed 34 critical genomic regions and 11 candidate genes, notably *CDH2*, *PARD3*, and *CFH*, which are linked to immune function and hormone regulation. Two key biological pathways—immune activation and pituitary development—were identified as central to cortisol production. These results underscore the potential for incorporating stress-resilience traits into dairy cattle breeding programs.

## 1. Introduction

Cortisol is a critical glucocorticoid hormone involved in the regulation of stress responses, immune function, and metabolic homeostasis in mammals [1]. In dairy cattle, cortisol plays a pivotal role in modulating physiological responses to environmental and pathogenic stressors, influencing animal health, welfare, and productivity [2]. Among the various stressors encountered by cattle, lipopolysaccharide (LPS) endotoxin represents a key microbe-associated molecular pattern (MAMP) that stimulates a potent innate immune response. LPS, a component of the outer membrane of Gram-negative bacteria, can induce systemic inflammation and has been implicated in conditions such as mastitis, acidosis, and septicemia [3,4,5]. Previously, we found that the administration of LPS as an experimental challenge elicits a dynamic physiological response, marked by elevated cortisol levels, increased body temperature, and changes in immune function, making it an important model for understanding stress-induced immune modulation in dairy heifers [6], cattle [7,8] and sheep [9].

Genetic variation in cortisol response has been of growing interest in dairy cattle research, as cortisol levels can influence susceptibility to disease, reproductive efficiency, and overall productivity [10]. Previous studies have explored the heritability of cortisol response in various livestock species, suggesting that stress responsiveness has a genetic basis and may be selectively modifiable [11]. For example, sheep research has shown that individual differences in cortisol response to bacterial endotoxin challenge are primarily determined by signaling within the hypothalamic–pituitary–adrenal axis [12]. However, limited research has examined the genetic architecture underlying cortisol response to LPS challenge in *Holstein heifers* and its potential correlations with economically important traits. Nanas et al. [13] identified thermotolerant cows with lower cortisol and HSP70 levels, suggesting an inherent genetic basis for heat stress resistance.

The present study aimed to address this knowledge gap by estimating the genetic parameters for serum cortisol response to LPS challenge in *Holstein heifers* and its linear associations with production, health, reproduction, and conformation traits, while identifying genomic regions and candidate genes associated with cortisol response via a genome-wide association study (GWAS). Understanding the genetic determinants of cortisol regulation could provide insights into the potential for selective breeding to enhance stress resilience and overall herd health. By integrating cortisol response data with genomic breeding values for key production, health, reproduction, and conformation traits, this study seeks to elucidate the genetic relationships between stress responsiveness and economically relevant characteristics in dairy cattle. The findings from this study may have important implications for advancing genetic selection strategies aimed at improving both animal health and welfare and productivity in the dairy industry.

## 2. Materials and Methods

### 2.1. Animals and LPS Challenge

A total of 252 *Holstein heifers* with a mean age at the sampling day of 245 ± 43 (min = 62, max = 365) were selected for this study during late summer. The heifers had an average body weight of 261 ± 38.7 kg. All animals were housed and managed at the Ontario Dairy Research Centre in Elora, Ontario, Canada, with all procedures conducted in compliance with the University of Guelph Animal Care Committee’s approved Animal Use Protocol (AUP #3436). All animals received 1 mL of 200 ng/kg of *Escherichia coli* LPS (O111: B4, Sigma-Aldrich, St. Louis, MO, USA) administered intramuscularly (trapezius muscle). Blood was collected by jugular venipuncture 4 h post-LPS administration in serum vacutainers and allowed to clot for 30 min at room temperature before centrifugation at 2500× *g* for 10 min for serum isolation, which was stored at −80 °C until further use.

### 2.2. Cortisol Analysis and Phenotyping

Total serum cortisol concentrations were measured using the competitive IMMULITE/IMMULITE 1000 Cortisol chemiluminescent enzyme immunoassay (Siemens Medical Solutions Diagnostics, Los Angeles, CA, USA) and analyzed with the IMMULITE 1000 analyzer (INTER MEDICO, Markham, ON, Canada), with an analytical sensitivity of 28 nmol/L.

### 2.3. Linear Association Analysis

To estimate the linear association between cortisol response and 55 Genomic Estimated Breeding Values (GEBVs), which were obtained from official national evaluations provided by the Canadian Dairy Network (now Lactanet), a linear regression model was fit as follows:

(1) $$y_{ijk}=\mu +{BY}_{i}+{SD}_{j}+\beta \times {GEBV}_{Trait,k}+e_{ijk}$$

where y_ijk_ is the cortisol response phenotype, μ is the overall mean, BY_i_ and SD_j_ are the fixed environmental effects of the ith Birth Year and jth Sampling Date, respectively, β is the coefficient of the fixed linear regression on trait GEBV of the kth animal (GEBV_Trait,k_) of one of the 55 different trait GEBV, and e_ijk_ is the random error term.

Fixed effects of Birth Year had 4 levels: 2018, 2019, 2020, and 2021, and Sampling Date had 22 levels, corresponding to LPS challenge sessions. All heifers were managed under standardized feeding and housing protocols at the Ontario Dairy Research Centre. Cortisol measurements were performed on a single IMMULITE 1000 analyzer by trained personnel using consistent procedures to control for operator-related variability.

Linear regressions of cortisol response on the 55 national-level GEBVs were used as a preliminary tool to explore potential associations between stress response and economically relevant traits.

### 2.4. Variance Components and Heritability Estimation

The software suite Blupf90+ v.2.57 [14,15] was used for the genomic analysis. Quality control was applied to keep SNP markers with allele frequencies higher than 0.01, call rates higher than 0.9, and animals with call rates higher than 0.9. Sex chromosomes were excluded from the analyses. Variance component and heritability estimation were performed using Residual Maximum Likelihood (REML) with the Average Information (AI) algorithm.

The linear mixed model used for variance component and GEBV estimation for cortisol response was the following:

(2) $$y_{ijk}=\mu +BY_{i}+SD_{j}+a_{k}+e_{ijk}$$

where y_ijk_ is the cortisol response phenotype, µ is the average, BY_i_ and SD_j_ are the fixed environmental effects of the ith Birth Year and jth Sampling Date, respectively, a_k_ is the random additive genetic effect of the kth animal, and e_ijk_ is the residual random error. a_k_ and e_ijk_ were assumed to follow a normal distribution with means equal to zero and covariance matrices equal to $G\sigma_{a}^{2}$ and $I\sigma_{e}^{2}$ respectively, where **G** is the genomic relationship matrix created as proposed by [16] and **I** is an identity matrix.

The heritability (h^2^) was estimated as:

(3) $$h^{2}=\frac{\sigma_{a}^{2}}{\left(\sigma_{a}^{2}+\sigma_{e}^{2}\right)}$$

The significance of the estimated additive genetic variance in the cortisol response was tested by a likelihood ratio test comparing the full model (2) vs. the reduced model without the animal additive genetic effect using the appropriate Chi-Squared test [17]. REML Multivariate normal sampling [18] was used to approximate the h^2^ estimate sampling error.

### 2.5. Single-Step Genome-Wide Association Studies (ssGWAS)

To perform the association study, heifers were classified into three groups based on cortisol concentrations using standard deviation thresholds from the population mean (573.4 nmol/L). High Responders (HRs) were defined as individuals with cortisol levels >956.0 nmol/L (mean + 1 SD), and Low Responders (LRs) as individuals with levels <190.8 nmol/L (mean − 1 SD). Medium Responders (MRs) fell within the range of ±1 SD from the mean. After this categorization process, there were 34 HR, 181 MR, and 37 LR animals. The phenotypes for all these 252 individuals were used in the analysis; however, to increase the statistical power to detect associations between the cortisol response and the markers, the genotypes of MR animals were removed, keeping only the genotypes of 71 animals (34 HR and 37 LR) in the ssGWAS analysis in Blupf90+ v.2.57 [14,15].

The model equation presented in the previous Equation (2) was also used in the ssGWAS analyses, where the covariance matrices of the vector of animal additive genetic effects (**a**) and the vector of residual random errors **e** were assumed to be equal to:

(4) $$var\left[\begin{array}{c} a\\ e \end{array}\right]=\left[\begin{array}{cc} H\sigma_{a}^{2} & 0\\ 0 & I\sigma_{e}^{2} \end{array}\right]$$

where the **H** is a hybrid relationship matrix that takes into account pedigree and genomic information [19], **I** is an identity matrix, $\sigma_{a}^{2}$ is the additive genetic variance, and $\sigma_{e}^{2}$ is the residual error variance. The **H^−1^** was obtained as below:

(5) $$H^{-1}=A^{-1}+\left[\begin{array}{cc} 0 & 0\\ 0 & G^{-1}-A_{22}^{-1} \end{array}\right]$$

where **G** was created as proposed by [16], and $A_{22}^{-1}$ represents the inverse of the pedigree relationship matrix for the genotyped animals.

Although population stratification was not explicitly modeled, the use of the H-matrix in ssGWAS reduces the likelihood of stratification bias due to genetic relationships.

After the GEBV prediction using Blupf90+, PostGSf90 was used to back-solve for the SNP effects and perform the GWAS. Two GWAS approaches were used; the first obtained the *p*-values of each individual SNP, which were used for identifying the significantly associated SNPs, and the second approach used windows of 10 SNPs to identify those that explained 0.5% or more of the genetic variation as significantly associated with cortisol response.

#### Gene and QTL Annotation

The results from both ssGWAS approaches, individual SNP tests and 10-SNP windows, were combined, and the most significant SNP (the smallest *p*-value) in each significant window (that explained 0.5% or more genetic variation) was used as a tag SNP for the windows. The tag SNP was used to perform the search for candidate genes and QTL, within a range of 50 kb upstream and downstream of the tag SNP, which was defined considering the average linkage disequilibrium for dairy cattle [20]. The candidate gene and QTL searches were performed using the R package GALLO v1.4 [21]. The same package was applied to perform a QTL enrichment analysis.

The online platform g:Profiler v.e112_eg59_p19_25aa4782 [22] was used to perform a functional enrichment analysis with the gene products found in the previous step. The online Reactome application [23] (Pathway Browser version 3.7 and Reactome database release 92) was used to generate an overall graphical illustration of the enriched pathways. The online tool STRING [24] v. 12.0 was used to check for protein–protein interactions in association network analysis.

## 3. Results

### 3.1. Variance Components and Heritability

The likelihood ratio test for the additive genetic variance was 2.45 (*p* = 0.059), which shows evidence of significant additive genetic variance in the cortisol response. The estimated additive genetic variance, phenotypic variance, residual variance, and heritability were 28,212, 105,350, 77,182, and 0.26 (SE: 0.19), respectively.

### 3.2. Linear Associations

Nine different traits showed significant (*p* < 0.05) or trending significant (*p* < 0.15) weak linear associations with cortisol response (Table 1). About 38% of the cortisol phenotype variance was explained by the models, which included BY and SD effects and the linear regression on the trait GEBV. As shown in Table 1, the absolute values of the linear correlation coefficients between cortisol levels and the trait EBVs varied from 0.10 to 0.18 for the nine traits.

### 3.3. ssGWAS

**Figure 1:**
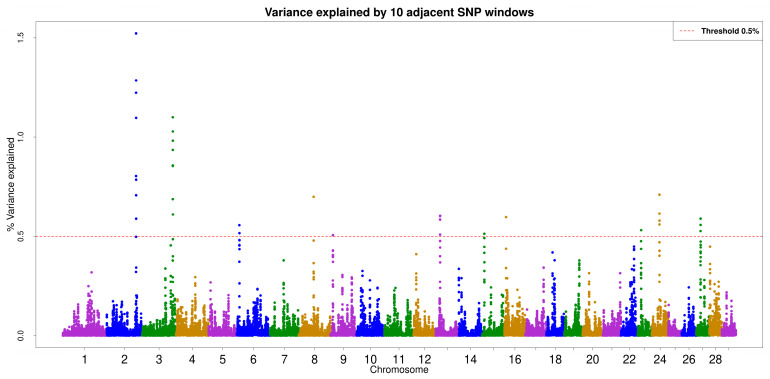
ssGWAS Manhattan plot for 10 adjacent SNP windows. Each dot represents the variance explained by each window. The dashed red line represents the threshold of 0.5% of additive genetic variance explained by a window. A total of 34 windows (dots) explained more than 0.5% of the additive genetic variance. These windows are located on chromosomes 2, 3, 6, 8, 9, 13, 15, 16, 23, 24, and 27.

After quality control, 42,123 SNPs were retained. Thirty-four 10-SNP windows explained at least 0.5% of the additive genetic variance. For these windows, the *p*-value for each SNP in the window was obtained (Supplementary Materials S1). Figure 1 depicts the Manhattan plot for the amount of additive genetic variance explained by the 10 SNPs in each window.

### 3.4. Gene and QTL Annotation

The search for genes in a 50 kb region surrounding the SNP with the smallest *p*-value from each window, which explained at least 0.5% of the additive genetic variance, found 11 different genes; of these, 9 encode proteins, 1 encodes lncRNA (long non-coding RNA), and 1 encodes miRNA (microRNA) (Table 2).

**Figure 2:**
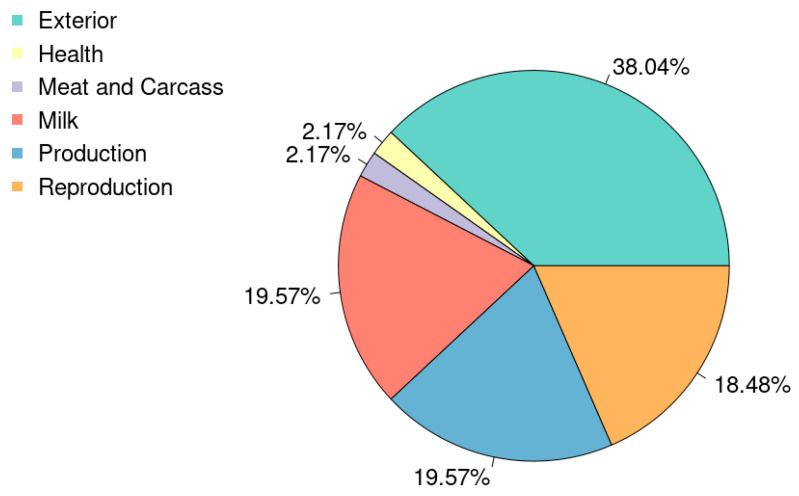
The percentage of QTLs by type found near the most significant SNP in each window that explained 0.5% or more of additive genetic variance.

The search for QTL returned 75 different QTL (Supplementary Materials S2). Figure 2 shows the QTL sorted by type, and Supplementary Materials S3 shows the detailed frequencies for each trait within the QTL type.

**Figure 3:**
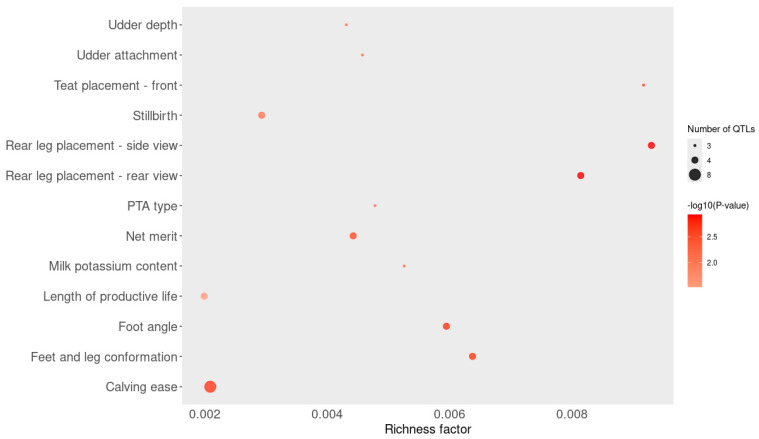
QTL enrichment plot for QTL close to the most significant SNP (50 kb upstream and downstream) in each window that explains 0.5% or more of additive genetic variance.

The enrichment analysis showed that 13 QTL are overrepresented in the sample investigated in this study (Figure 3 and Supplementary Materials S4).

### 3.5. Functional Enrichment Analysis

The gene enrichment analysis revealed 17 enriched terms that are presented in Table 3, 6 of which are molecular functions and 2 are cellular components. Supplementary Materials S5 display the enriched pathways, only for Reactome analysis, clustered in cell–cell communication, developmental biology, the immune system, and signal transduction.

The only observed interaction among gene products near the most significant SNPs was between the *PARD3* and *CDH2* genes (Supplementary Materials S6). Notably, these two genes were also the only ones associated with overlapping enriched terms, as detailed in Table 3.

## 4. Discussion

### 4.1. Linear Association Between Cortisol Response and Important Genetically Evaluated Traits

Significant but weak linear associations of cortisol response with GEBV were found for several traits evaluated in Canada. Some associated traits are directly related to milk production, such as milk yield and protein yield, and some are closely related to milk production, such as udder texture, milking speed, and lactation persistency.

Milk-related traits have several pieces of evidence of association with cortisol levels in the literature. Cortisol concentration in milk has been found to vary with lactation stage, parity, and milk production level in goats [25]. In dairy cows, hair cortisol concentration showed positive correlations with cumulative milk yield, milk urea concentration, and somatic cell count, suggesting an association with metabolic stress and inflammation during lactation [26]. Milk cortisol levels were found to be highest during the first week of lactation in cows and remained stable thereafter. Cortisol concentrations in milk can serve as a biomarker for short-term stress responses, with levels correlating to plasma cortisol [27]. While elevated cortisol can reduce mammary tight junction permeability, potentially preventing milk loss due to leakiness, it may also decrease milk yield by impairing the uptake of key metabolic precursors, such as glucose and amino acids, which are essential for lactose and milk protein synthesis in the mammary gland [27]. Other traits found to be correlated with cortisol response in this study have also been previously related to cortisol level, like energetic metabolism traits, such as body maintenance requirement and clinical ketosis [28,29], and related to animal health, such as cystic ovaries [30,31] and heel horn erosion [32,33].

Interestingly, our study found no significant association between cortisol response and somatic cell score (SCS), an established indicator of udder inflammation and mastitis susceptibility. This stands in contrast to previous observations in goats, where ACTH-induced cortisol elevations were associated with increased milk SCC [34]. The absence of a detectable correlation in our dataset may reflect key differences between acute HPA axis activation by LPS versus chronic or localized mammary inflammation, which SCS typically represents. These processes likely involve distinct regulatory pathways and gene networks. From a livestock breeding perspective, however, the lack of an antagonistic association between cortisol responsiveness and SCS is promising, implying that selection for stress resilience could proceed without compromising udder health. Future research, including longitudinal cortisol and SCS measurements across all response phenotypes, is warranted on other populations to better understand the relationship between HPA activation and mastitis susceptibility.

Previous studies have demonstrated that cortisol production varies significantly among individuals, with high heritability estimates reported in pigs following adrenocorticotropic hormone (ACTH) stimulation (heritability ≈ 0.64), indicating strong genetic control of HPA axis responsiveness [11,35] depending on the stress challenge. This inter-individual genetic variability forms a plausible basis for linking cortisol to complex traits such as metabolic efficiency and reproductive performance. Mormède et al. [36] and Hough et al. [37] argued that cortisol-related responses are key to defining animal robustness, particularly under fluctuating environmental conditions.

### 4.2. Candidate Genes and Enriched Terms

**Figure 4:**
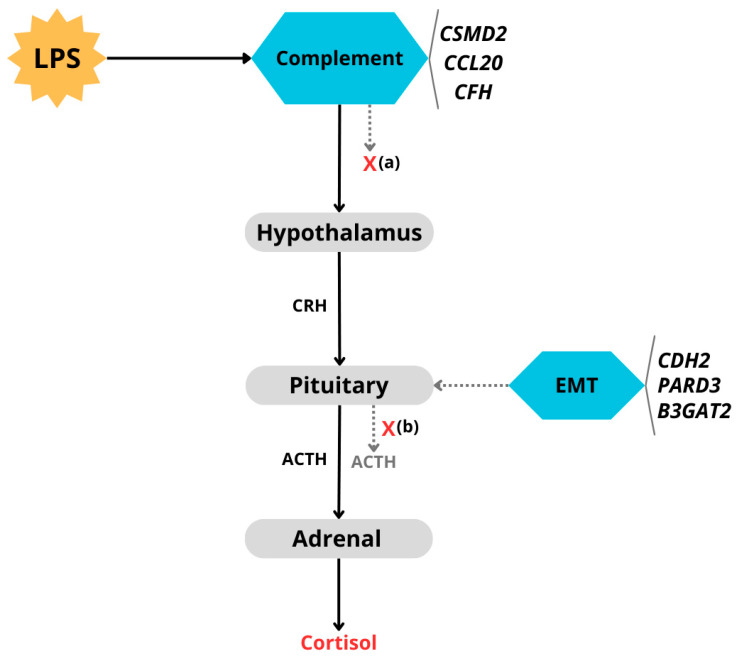
Possible influence of candidate genes in cortisol production via the HPA axis. LPS: Lipopolysaccharide, (**a**) possible interference (X) in hypothalamus stimulation via genes involved in the complement system; (**b**) possible influence (X) in pituitary gland development and ACTH production by CDH2 variant via interference in EMT (Epithelium–Mesenchymal Transition) cellular process.

Cortisol is a hormone primarily synthesized in the adrenal gland in response to hypothalamic–pituitary–adrenal (HPA) axis activation. Briefly, when the organism is under stress, the hypothalamus will release corticotropin-releasing hormone (CRH), which stimulates the pituitary gland to liberate adrenocorticotropic hormone (ACTH) into the bloodstream. This hormone will stimulate cortisol production and release by the adrenal gland (Figure 4), so that cortisol can carry out its several functions, for instance, glucose metabolism, stress response, and inflammation suppression. High cortisol levels will work as negative feedback to the hypothalamus and pituitary to inhibit CRH and ACTH production, respectively [38].

In this study, eight candidate genes—*CSMD2*, *DAW1*, *HMGB4*, *B3GAT2*, *CCL20*, *CFH*, *PARD3* ,and *CDH2*—were identified close to markers significantly associated with the cortisol response in *Holstein heifers* following an LPS challenge. These genes are associated with innate immunity, cell–cell communication, and adhesion.

Based on the functions and pathways associated with the candidate genes, two primary mechanisms were inferred through which cortisol production via the HPA axis may be modulated. As illustrated in Figure 4, the first mechanism (labeled “a”) involves the activation and regulation of the complement system, in which several candidate genes play a role. The second mechanism (labeled “b”) involves genetic variants in *CDH2*, *PARD3*, and *B3GAT2*, which may interfere with epithelial–mesenchymal transition (EMT), potentially leading to abnormal pituitary gland development. This could result in impaired production of hormones such as ACTH, ultimately affecting cortisol synthesis. While mechanism (b) may affect cortisol production irrespective of the stimulus source—due to its direct involvement in pituitary gland function—mechanism (a) appears to be specific to the nature of the stimulus used in this study (i.e., LPS), which activates the complement system, a central component of the innate immune response.

Epithelial–mesenchymal transition (EMT) is a cellular process in which epithelial cells transform into mesenchymal cells, characterized by two key changes: the loss of apicobasal polarity and the disruption of cell–cell adhesion. These changes grant the cells increased mobility and invasiveness and are particularly important during embryonic development [39]. In this context, two candidate gene products may play significant roles: PARD3, a component of the Par complex involved in establishing apicobasal polarity [40]; and CDH2, which regulates cell–cell communication, a critical process for adhesion, cell sorting, and tissue morphogenesis [41].

The anterior pituitary, responsible for the synthesis and release of peptide hormones, including ACTH produced by corticotrophs, develops from Rathke’s pouch—an invagination of oral ectoderm—during fetal development [42]. The transformation of the more columnar epithelial cells in Rathke’s pouch into the rounded, dispersed cells found in the anterior lobe resembles the EMT process [43], suggesting a potential link between EMT regulation and proper pituitary gland development.

CDH2 (also known as N-cadherin) is a calcium-dependent cell adhesion protein that mediates homophilic cell–cell adhesion through dimerization with CDH2 chains on adjacent cells [44]. It plays a central role in the formation of adherens junctions by linking the actin cytoskeleton to the cell membrane through its cytoplasmic domain, which interacts with α- and β-catenins, as well as p120-catenin [41]. In a study by Himes et al. [41], CDH2 was shown to be essential for pituitary gland organogenesis and homeostasis in mice. Specifically, it was critical for maintaining the spatial organization of anterior pituitary cells, including corticotrophs responsible for ACTH production.

In humans, Ferreira et al. [45] reported a case involving a patient with an ectopic posterior pituitary lobe and deficiencies in several hormones, including growth hormone (GH), thyroid-stimulating hormone (TSH), ACTH, and prolactin (PRL). Genetic analysis revealed that the patient was homozygous for a CDH2 variant (G > A at position 865), resulting in a missense mutation (p.Val289Ile). In contrast, her unaffected siblings and parents were heterozygous for this variant. The p.Val289Ile substitution was associated with reduced homophilic adhesion capacity of N-cadherin, leading to smaller and less cohesive cell aggregates. The authors suggested that this mutation may impair adenohypophyseal placode development by disrupting normal cell migration, ultimately contributing to morphological abnormalities of the pituitary gland and hormone deficiencies, including reduced ACTH levels.

As shown in Table 3, CDH2 and PARD3 were jointly associated with four enriched functional terms: adherens junction, cell junction organization, cell–cell junction organization, and cell–cell communication. Additionally, CDH2 was linked to four more terms (gamma-catenin binding, alpha-catenin binding, myogenesis, and interhypothalamic adhesion), while PARD3 appeared in three others (PAR polarity complex, tight junction interactions, and TGF-beta receptor signaling in EMT). Together, these two genes were involved in 11 of the 17 enriched terms identified, most of which are directly related to cell–cell communication, polarity, and junctional organization. Furthermore, their gene products were found to interact in the network analysis (Supplementary Materials S6), reinforcing their functional connection.

The presence of *PARD3* and *CDH2* genes in common cellular processes has already been demonstrated. During Zebrafish embryo development, the spatial deployment of the PARD3 and CDH2e proteins is modified during the de novo polarization of the epithelial tube [46]; also in Zebrafish, Guo et al. [47] showed that *PARD3* and *CDH2* genes participate in different stages of neural tube formation. While CDH2 is considered a pioneer protein, PARD3’s role is related to the intermediate stage of neurulation. *PARD3* and *CDH2* gene participation in a common process reinforces even more the mutual presence of these two genes in enriched terms. *B3GAT2* (beta-1,3-glucuronyltransferase 2) gene is annotated to the enriched GO term galactosylgalactosylxylosylprote 3-beta-glucuronosyltransferase activity, its product participates in HNK-1 (human natural killer-1) synthesis, which is involved in cell migration and adhesion [48]. This GO term is a glucuronosyltransferase activity [49], which plays an important role in the pathway for Glycosaminoglycan (GAG) synthesis [50] and formation of proteoglycan (PT), one of the main compounds of the Extracellular Matrix (ECM) [51]. Since GAG and PT biosynthesis are essential for ECM (Extracellular Matrix) structure and function, *B3GAT2* could also be associated with the instance “(b)”, as Kim et al. [52] highlighted that with the transition from epithelial to mesenchymal cells, the interaction of epithelial cells with the basement membrane is lost, and then the mesenchymal cells start an interaction with the Extracellular Matrix (ECM). In summary, the *CDH2*, *PARD3*, and *B3GAT2* genes seem to be associated with the EMT process, which may be related to pituitary gland development. Atypical pituitary gland morphology and *CDH2* mutation were already related to deficiency in ACTH levels, so, considering the HPA axis, it is reasonable to consider these three genes as candidate genes for cortisol response to LPS challenge.

As shown in Table 3 and Supplementary Table S5, another group of enriched functional categories, cellular components, and pathways is associated with the immune system, particularly the complement system. The genes *CSMD2* and *CFH* were identified as contributors to these immune-related functions, which are represented as instance “(a)” in Figure 4. *CSMD2* (CUB and Sushi Multiple Domains 2) gene plays a role in the complement system, a key component of innate immunity that facilitates pathogen detection and inflammation regulation [53,54]. Inflammation activates the HPA axis, leading to increased cortisol secretion [55,56]. CFH (complement factor H) is a soluble regulator involved in complement regulation, acting in complement component C3b inactivation and avoiding C3 and C5 convertase formation, hindering additional activation of the complement cascade [57,58]. Both gene products (CSMD2 and CFH) may contribute to the observed variation in cortisol response by modulating the inflammatory response initiated by LPS challenge. Notably, complement system dysregulation has been linked to stress responses in other species [59,60,61], suggesting a conserved mechanism across mammals. The final enriched pathway identified was EBV LMP1 signaling, which includes the gene *CCL20*. This gene encodes the chemokine macrophage inflammatory protein-3α (MIP-3α or CCL20), which plays a key role in directing the migration of immune cells expressing the CCR6 receptor, such as dendritic cells, B cells, and both T helper 17 and regulatory T cells [62]. In humans, CCL20 is highly expressed in immune-relevant tissues, including the lungs, intestines, liver, thymus, skin, prostate, and testis [63]. Circulating CCL20 has been proposed as a biomarker of inflammation in various conditions, including neurological disorders [62], arthritis [64,65], and endotoxemia and hepatitis [66]. In dairy cattle, Brand et al. [67] reported CCL20 expressed in the mammary gland following LPS perfusion, while Jiang et al. [68] observed its expression in rumen epithelial cells stimulated with LPS, supporting its involvement in bovine immune responses.

Based on the enriched terms, two distinct mechanisms emerge through which cortisol production in response to LPS stimulation may be influenced: (1) via the complement system and innate immune response, and (2) via epithelial–mesenchymal transition (EMT) and pituitary gland organogenesis. The significance of these two pathways is further highlighted in Supplementary Materials S5, where the clusters related to immune system, developmental biology, and cell–cell communication encompassed nearly all identified enriched terms.

For the two remaining genes, *DAW1* and *HMGB4*, no direct evidence was found that would link them with the immune system or pituitary gland organogenesis. *DAW1* (Dynein Arm Deficiency 1) is primarily involved in spindle formation during cell division, contributing to tissue repair and regeneration [69]. While DAW1’s direct role in immune or stress responses is less well-documented, its function in cellular proliferation suggests it may support recovery from stress-induced tissue damage [70]. Increased cellular turnover could be particularly relevant in epithelial tissues exposed to inflammatory insults following an LPS challenge, where effective repair mechanisms help restore homeostasis.

The *HMGB4* gene codes for one of four high-mobility group box proteins that have critical roles in regulating chromatin architecture, DNA replication and repair, and gene transcription [71]. Expression of these HMGBs is diverse across species and tissues; HMGB4 appears unique to mammals. In humans, HMGB4 is expressed in the embryonic pancreas and brain, implicating its role in organ development. During adulthood, HMGB4 is localized in neural cells and the testis [72]; additionally, expression of HMGB4 has recently been implicated in male infertility [73]. When HMGBs translocate from the nucleus to the cytoplasm, they trigger programmed cell death. Cytoplasmic leakage of HMGBs can trigger systemic inflammation that can lead to sepsis because oxidized HMGBs are recognized as danger signals (alarmins) by various cells of the immune system [74]. Although studies involving HMGB4 are limited, serum levels of HMGB1 have been positively associated with the severity of several inflammatory diseases [75,76,77].

Beyond the mechanisms outlined in Figure 4, *PARD3* may also contribute to epithelial barrier integrity by promoting tight junction assembly in epithelial tissues, thereby preserving tissue homeostasis and preventing pathogen translocation [78,79]. In cattle, compromised barriers—in the gut, mammary gland, or respiratory epithelium—can precipitate localized inflammation, systemic immune activation, and stress responses [80,81]. Because epithelial injury can drive pro-inflammatory signaling that, in turn, stimulates the HPA axis and elevates cortisol levels, PARD3-mediated maintenance of tight junctions may serve a protective role by limiting excessive cortisol release.

The functional enrichment analysis identified genes near significant SNPs that are involved in key biological processes relevant to cortisol response following LPS challenge. Notably, enrichment of immune-related functions, such as complement binding, C3b binding, and opsonin binding, was largely driven by CFH, a regulator of the complement system. This supports the role of cortisol in the innate immune modulation and its role in inflammatory regulation. Genes such as *CDH2*, *PARD3*, and *B3GAT2* were associated with enriched terms related to adherens and tight junctions, cell–cell communication, cell polarity, Extracellular Matrix, and epithelial integrity, which can be associated with the EMT process and pituitary gland organogenesis. The CCL20 is known to be secreted by mammary epithelial cells in response to bacterial challenge [82] and plays a role in recruiting immune cells to the mammary gland [83]. Overall, these findings suggest that cortisol response is influenced by genes regulating immune activity, epithelial barrier function, and neuroendocrine signaling, offering new insights into the genetic control of stress resilience in dairy cattle. Together, these candidate genes echo multi-systemic effects of cortisol that were highlighted in omics-based research showing cortisol-related methylation and gene expression variations in both immune and neuroendocrine pathways [84,85].

The QTL enrichment analysis (Figure 3) provides further insight into the genetic architecture underlying cortisol response in *Holstein heifers*. Surrounding the most significant SNP in genomic windows explaining ≥0.5% of the additive genetic variance, QTLs associated with milk production, udder morphology, reproductive traits, and productive life were significantly overrepresented. This enrichment suggests a potential pleiotropic relationship or close genomic linkage between loci involved in stress hormone regulation and those influencing key performance traits. Notably, traits such as udder texture, teat placement, and calving ease, often targeted in breeding programs, may share underlying genetic mechanisms with cortisol responsiveness. These findings support the hypothesis that animals with distinct stress response profiles may differ not only in immune function but also in productive and reproductive performance. Incorporating cortisol-associated genomic information into breeding programs may thus offer a strategy to improve both resilience and production in dairy cattle.

## 5. Conclusions

This study provides novel insights into the genetic architecture of cortisol response in *Holstein heifers* following an LPS challenge. A significant estimated additive genetic variance, along with moderate heritability of cortisol response, suggests that individual variation in cortisol response has a genetic basis and could be exploited in selective breeding. Weak, significant linear associations between cortisol response and key GEBVs, including milk yield, protein yield, body maintenance, lactation persistency, and cystic ovaries, highlighted the prospective physiological and economic relevance of cortisol response. Multiple genomic regions and candidate genes were identified that could interfere with cortisol production in two distinct ways associated with the HPA axis: the first related to innate immune and complement systems, where the genes *CFH*, *CCL20*, and *CSMD2* are involved, and the second associated with the EMT process during pituitary gland formation, where the genes *CDH2*, *PARD3*, and *B3GAT2* could play a role. Enrichment analyses further revealed overlapping genetic control between cortisol response and traits related to milk production, udder conformation, fertility, and productive life. These findings suggest that integrating stress responsiveness into breeding programs could enhance animal health, resilience, and overall productivity. Future research using larger datasets and functional validation of candidate genes will be essential to translate these findings into practical applications for dairy cattle breeding.

## Figures and Tables

**Table 1:** Linear associations of cortisol levels with genomic estimated breeding value of several traits.

Trait	$\mathrm{Model}\;R^{2}$	Model F-Value	Model *p*_Value	β	*ρ*	*p*-Value
CO	0.391	5.967	1.72 × 10^−14^	−11.185	−0.178	0.010
BMR	0.390	5.931	2.17 × 10^−14^	14.461	0.173	0.014
LP	0.386	5.830	4.19 × 10^−14^	12.262	0.145	0.033
MILK	0.385	5.810	4.79 × 10^−14^	0.067	0.144	0.040
PROT	0.384	5.789	5.46 × 10^−14^	2.217	0.137	0.048
UT	0.382	5.743	7.38 × 10^−14^	−12.124	−0.125	0.073
CK	0.380	5.692	1.03 × 10^−13^	12.733	0.113	0.119
HHE	0.380	5.677	1.13 × 10^−13^	7.400	0.103	0.139
MSP	0.379	5.671	1.18 × 10^−13^	−7.239	−0.100	0.148

CO: cystic ovaries; BMR: body maintenance requirements; LP: lactation persistency; MILK: milk yield; PROT: protein yield; UT: udder texture; CK: clinical ketosis; HHE: heel horn erosion; MSP: milking speed. R^2^: Coefficient of determination of the full model. β: Linear coefficient of the regression of cortisol response on the trait GEBV. *ρ*: correlation coefficient between cortisol response and the trait GEBV.

**Table 2:** Genes surrounding (50 kb upstream and downstream) the most significant SNP in the windows that explain 0.5% or more of additive genetic variance.

SNP	rsID	chr	Start_Pos	End_Pos	Gene_id	Gene_Name	Gene_Biotype
ARS-BFGL-NGS-43721	rs108974471	2	115948258	115951955	ENSBTAG00000021326	CCL20	protein_coding
ARS-BFGL-NGS-43721	rs108974471	2	115992779	116028253	ENSBTAG00000021327	DAW1	protein_coding
ARS-BFGL-NGS-107330	rs109766798	2	115992779	116028253	ENSBTAG00000021327	DAW1	protein_coding
ARS-BFGL-NGS-25298	rs109868537	3	111603940	112289188	ENSBTAG00000005784	CSMD2	protein_coding
ARS-BFGL-NGS-85333	rs110742206	3	111603940	112289188	ENSBTAG00000005784	CSMD2	protein_coding
ARS-BFGL-NGS-110683	rs110606737	3	111603940	112289188	ENSBTAG00000005784	CSMD2	protein_coding
ARS-BFGL-NGS-85333	rs110742206	3	111927003	111927727	ENSBTAG00000000335	HMGB4	protein_coding
ARS-BFGL-NGS-57285	rs109872657	9	9970661	10070666	ENSBTAG00000020817	B3GAT2	protein_coding
BTA-25900-no-rs	rs41575397	13	18449975	18466359	ENSBTAG00000052242	NA	lncRNA
BTA-25900-no-rs	rs41575397	13	18484975	19062784	ENSBTAG00000014991	PARD3	protein_coding
ARS-BFGL-NGS-13518	rs110188001	13	18484975	19062784	ENSBTAG00000014991	PARD3	protein_coding
ARS-BFGL-NGS-109707	rs109869165	13	18484975	19062784	ENSBTAG00000014991	PARD3	protein_coding
ARS-BFGL-NGS-13518	rs110188001	13	18847324	18847381	ENSBTAG00000054243	bta-mir-2285aw	miRNA
ARS-BFGL-NGS-109707	rs109869165	13	18847324	18847381	ENSBTAG00000054243	bta-mir-2285aw	miRNA
BTB-01975868	rs43082091	16	6199772	6333310	ENSBTAG00000023177	CFH	protein_coding
ARS-BFGL-NGS-118806	rs42048457	24	28655047	28904115	ENSBTAG00000021190	CDH2	protein_coding
BTB-00952622	rs42110734	27	16673409	16675767	ENSBTAG00000050498	NA	protein_coding
ARS-BFGL-NGS-58358	rs109507088	27	16673409	16675767	ENSBTAG00000050498	NA	protein_coding

**Table 3:** Functional enriched terms for the gene products found close to the most significant SNP (50 kb upstream and downstream) in each window that explain 0.5% or more of additive genetic variance.

Source	Term Name	Term Id	*p*-Value *	Intersections	
GO:MF	galactosylgalactosylxylosylprotein 3-beta-glucuronosyltransferase activity	GO:0015018	0.042	B3GAT2	
GO:MF	gamma-catenin binding	GO:0045295	0.048	CDH2	
GO:MF	complement component C3b binding	GO:0001851	0.048	CFH	
GO:MF	complement binding	GO:0001848	0.048	CFH	
GO:MF	alpha-catenin binding	GO:0045294	0.048	CDH2	
GO:MF	opsonin binding	GO:0001846	0.048	CFH	
GO:CC	adherens junction	GO:0005912	0.048	PARD3	CDH2
GO:CC	PAR polarity complex	GO:0120157	0.048	PARD3	
REAC	cell–cell junction organization	REAC: R-BTA-421270	0.005	PARD3	CDH2
REAC	cell junction organization	REAC: R-BTA-446728	0.006	PARD3	CDH2
REAC	cell–Cell communication	REAC: R-BTA-1500931	0.006	PARD3	CDH2
REAC	tight junction interactions	REAC: R-BTA-420029	0.020	PARD3	
REAC	TGF-beta receptor signaling in EMT (epithelial to mesenchymal transition)	REAC: R-BTA-2173791	0.039	PARD3	
REAC	myogenesis	REAC: R-BTA-525793	0.048	CDH2	
WP	EBV LMP1 signaling	WP:WP984	0.032	CCL20	
WP	complement and coagulation cascades	WP:WP1056	0.038	CFH	
HP	interhypothalamic adhesion	HP:0033105	0.045	CDH2	

GO: Gene Ontology; MF: Molecular Function; CC: Cellular Component; WP: WikiPathways; HP: Human Phenotype Ontology. * Benjamini and Hochberg false discovery rate threshold. Intersections: genes shared between the input query list and a given functional term.

## Data Availability

The datasets used and analyzed during the current study are available from the corresponding author upon request.

## Supplementary Materials

The following supporting information can be downloaded at: https://www.mdpi.com/article/10.3390/ani15131890/s1, Table S1: 10-SNP windows with individual SNP tests and variance explained by each windows; Table S2: QTLs close to the most significant SNP in each significant windows; Figure S3: Percentage of each trait annotated as a production, reproduction, milk, meat and carcass, health and exterior QTLs found close to the most significant SNP in each window that explained 0.5% or more of additive genetic variance; Table S4: Enriched QTLs close to the most significant SNP in each significant windows; Figure S5: Reactome pathway enrichment map highlighting biological pathways associated with gene products located close to the most significant SNP (50 kb upstream and downstream) in each window that explain 0.5% or more of additive genetic variance. Pathway analysis was performed using Reactome (database release 92, pathway browser version 3.7). Figure S6: Protein–protein interaction (PPI) network of gene products encoded by genes located close to the most significant SNP (50 kb upstream and downstream) in each window that explain 0.5% or more of additive genetic variance. In the image, a notable connection between PARD3 and CDH2 genes is observed, which may reflect coordinated roles in cell polarity and adhesion.
