# Supplementary material for: Genetic Parameters, Linear Associations, and Genome-Wide Association Study for Endotoxin-Induced Cortisol Response in Holstein heifers
Source: Animals (Basel). 2025 Jun 26;15(13):1890. doi: 10.3390/ani15131890 (PMC12248804; doi:10.3390/ani15131890)
Supplement: Supplementary file 1 [file animals-15-01890-s001.zip › S3_Figure_qtl_names.pdf]

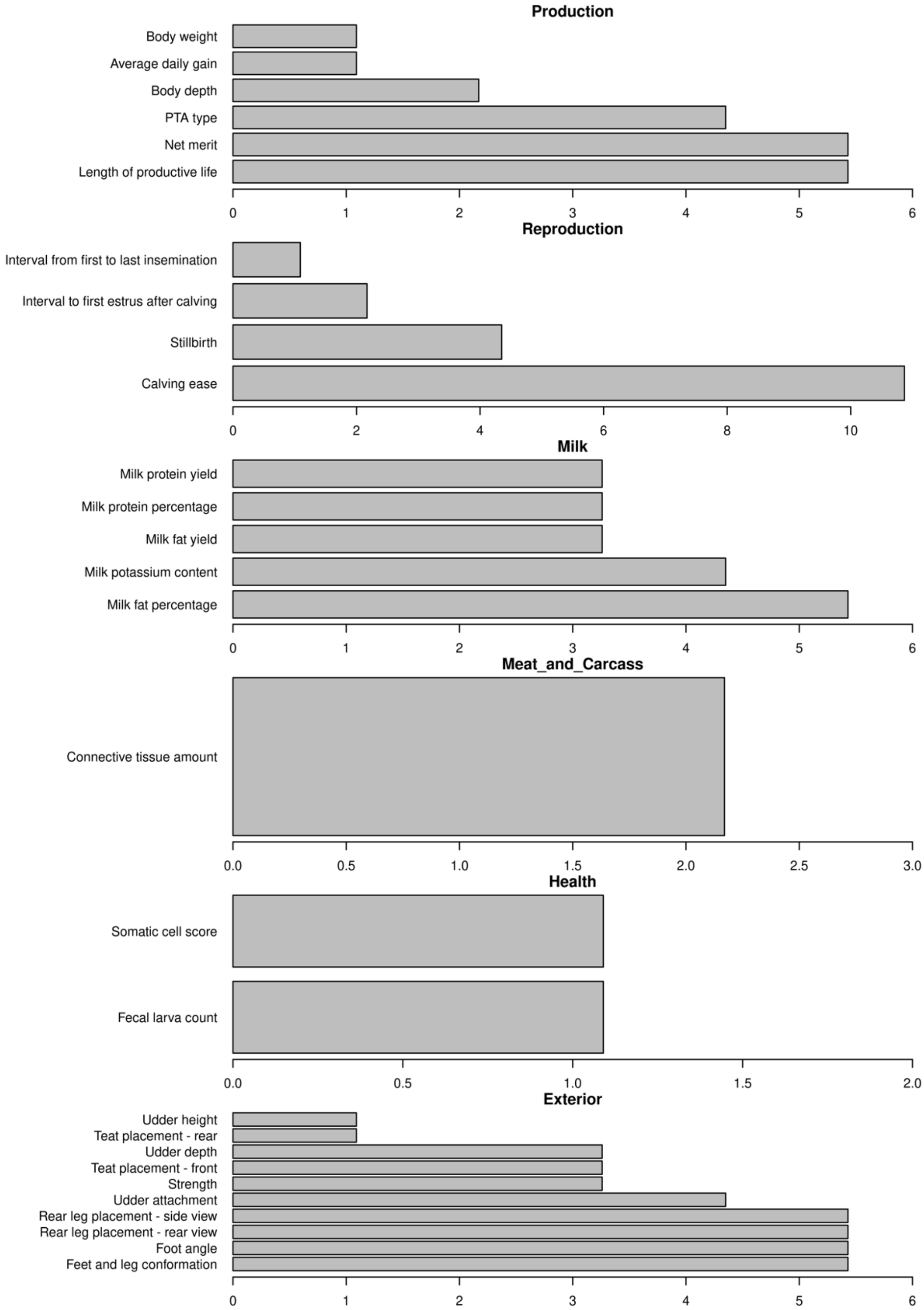

Figure S3 - Percentage of each trait annotated as a production, reproduction, milk, meat and carcass, health and exterior QTLs found close to the most significant SNP in each window that explained 0.5% or more of additive genetic variance
