# Supplementary material for: Genetic Parameters, Linear Associations, and Genome-Wide Association Study for Endotoxin-Induced Cortisol Response in Holstein heifers
Source: Animals (Basel). 2025 Jun 26;15(13):1890. doi: 10.3390/ani15131890 (PMC12248804; doi:10.3390/ani15131890)
Supplement: Supplementary file 1 [file animals-15-01890-s001.zip › S5 - Reactome_Enrichment_plot.pdf]

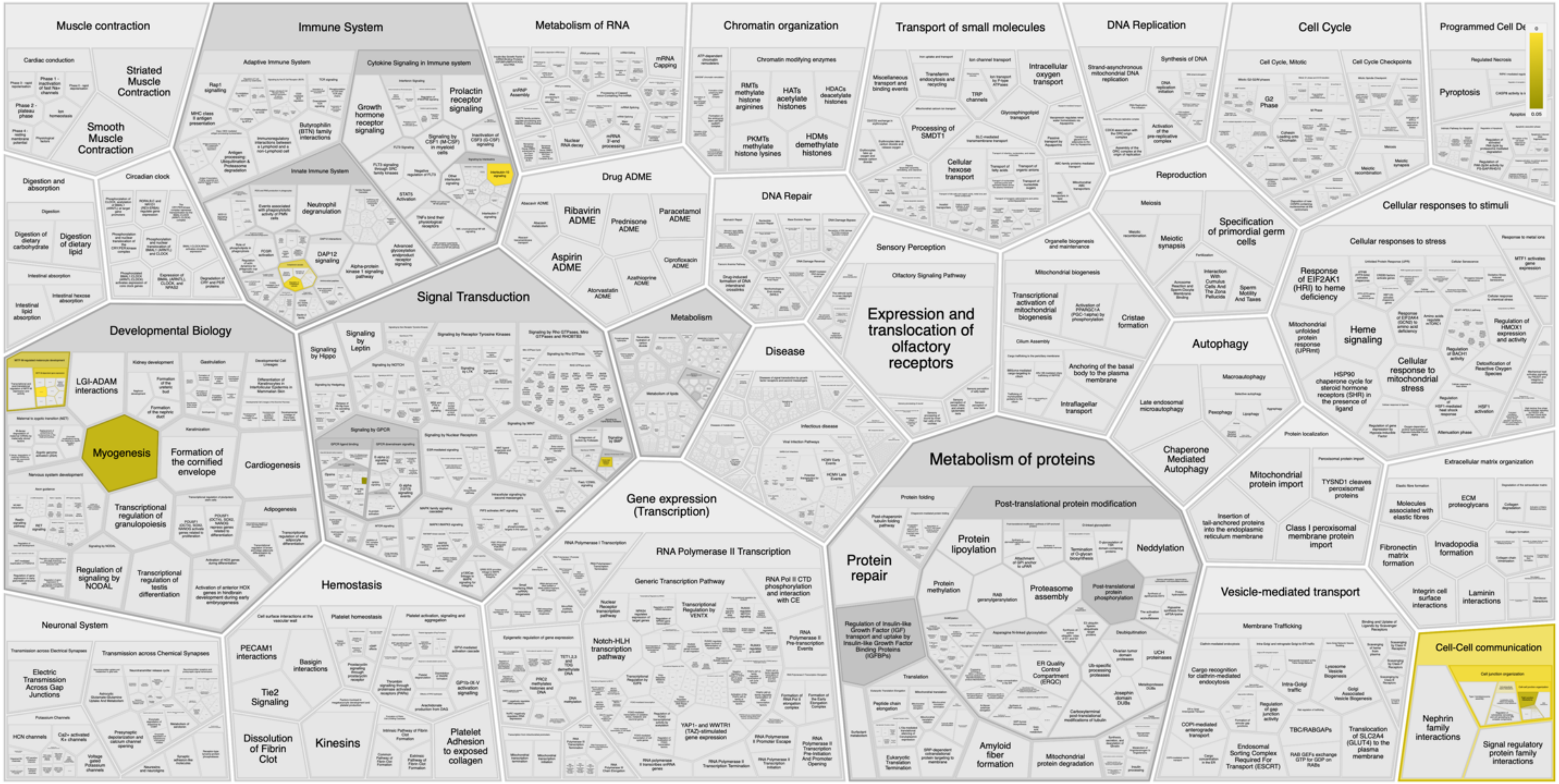

Figure S5 – Reactome pathway enrichment map highlighting biological pathways associated with gene products located close to the most significant SNP (50kb upstream and downstream) in each window that explain 0.5% or more of additive genetic variance. Pathway analysis was performed using Reactome (database release 92, pathway browser version 3.7).
